# Long-Term Follow-Up of Legacy Services Offered by Children's Hospitals in the United States

**DOI:** 10.1089/pmr.2021.0009

**Published:** 2021-08-13

**Authors:** Terrah Foster Akard, Samantha Burley, Maggie C. Root, Mary S. Dietrich, Brittany Cowfer, Kim Mooney-Doyle

**Affiliations:** ^1^ Vanderbilt University School of Nursing, Nashville, Tennessee, USA.; ^2^ University of Maryland School of Nursing, Baltimore, Maryland, USA.; ^3^ Vanderbilt Children's Hospital, Nashville, Tennessee, USA.; ^4^ University of Maryland School of Nursing, Baltimore, Maryland, USA.

**Keywords:** hospice and palliative care nursing, hospitalized child, palliative care, palliative medicine, survey, terminal care

## Abstract

***Background:*** Our 2012 survey of providers described legacy services offered at children's hospitals nationwide. Since then, the science related to legacy interventions has advanced, resulting in increased recognition of the importance of legacy services. Yet, legacy interventions offered by children's hospitals have not been recently described.

***Objective:*** To describe current legacy services offered by children's hospitals in the United States and compare with our previous results.

***Design:*** Descriptive cross-sectional design.

***Setting/Subjects:*** Participants included providers (*N* = 54) from teaching children's hospitals in the United States.

***Measurements:*** Electronic REDCap survey.

***Results:*** Similar to our prior research, 100% of respondents reported that their hospital offers legacy activities with 98% providing such services as a standard of care. Notable increased numbers of children are participating in legacy interventions compared with the previous study, now with 40% (compared with 9.5% previously) of participants reporting >50 children per year. Patients being offered legacy activities include neonatal intensive care unit (NICU) patients, those with life-threatening traumatic injuries, those on life support for extended periods of time, and those referred to hospice. Although not statistically significant, the percentage of hospitals offering legacy-making to children with cancer, neurodegenerative diseases, and life-threatening illnesses is slightly increased from the prior time point.

***Conclusions:*** Children across developmental stages and illness contexts and their families can benefit from both the memories generated through the process of legacy services and the subsequent tangible products. Providers should continue to offer legacy opportunities to seriously ill children and their families across a wide array of settings and illness contexts.

## Introduction

Nearly 4 million children worldwide are estimated to need pediatric palliative care,^1^ and ∼17 million parents are caring for children with serious illness in the United States.^2^ In addition to substantial physical symptom distress,^3–5^ children with serious illness have shown high levels of depression, sadness, distress, anxiety, and worry.^3,5,6^ Children may question God and struggle with lack of meaning for their illness and concern for loved ones they will leave behind as they prepare for their impending deaths.^7–9^ Parents may suffer anxiety, depression, marital disruptions, job loss, family financial strain, and below standard quality of life.^4,10–15^

Legacy-making, actions or behaviors aimed at being remembered, is one strategy to improve child and parent psychosocial distress during advanced illness for patients and their families.^16–20^ Leaving a legacy can be a concern for children with serious illness who are developmentally able to understand that death is permanent and irreversible and likely to think about death even if they do not communicate it explicitly.^20^ For children whose deaths can be anticipated, efforts to create memories and confirm they are loved and will be remembered are important.^21^ For example, some children delegate who will receive certain belongings after their death, write letters, draw pictures, take a special trip, or speak with significant people.^20^ In turn, such activities may facilitate documentation of children's legacies. Hospital staff have reported that legacy activities helped ill children cope and communicate and family members cope, communicate, and continue bonds in the case of the child's death.^16^

Legacy activities have been explored in both adult and pediatric populations.^16,17,19,20,22–31^ Legacy interventions in adults have been shown to increase patients' sense of dignity, purpose, meaning, and will to live, whereas decreasing suffering and depressive symptoms.^24,27,31^ Legacy interventions in children with cancer has shown promise to improve emotional quality of life, communication, and coping among children with cancer (aged 7–17 years).^16,22,23,32,33^ Parents have reported that legacy interventions improved parent–child communication (72%, *n* = 57), parent emotional comfort (63%, *n* = 50), parent coping (46%, *n* = 36), child expression of feelings (86%, *n* = 70), and child emotions (59%, *n* = 48).^22^

Our previous national survey of providers described legacy services offered at children's hospitals in the United States.^16^ Since that survey in 2012, research related to legacy interventions has advanced,^17,22,23,32–34^ as well as availability, awareness, and acceptability of legacy services. As a result, legacy interventions and services as a component of palliative care has received increased recognition. For example, providers have recommended memory-making and legacy-building as a priority domain for quality pediatric home-based palliative care.^35^ However, gaps persist for recent literature to describe current services offered to hospitalized children with serious illnesses and their families. Updating our previous survey from 2012 is necessary to (1) conduct a long-term comparison of legacy services offered to children with serious illness, (2) identify advances in care related to legacy services provided to children with serious illness, and (3) identify remaining gaps in care related to legacy services. Thus, the purpose of this study was to describe current legacy services offered by children's hospitals in the United States and compare with our 2012 results.

## Methods

Provider reports of legacy activities were collected using the same electronic REDCap survey developed for our original study.^16^ REDCap is a secure web platform for building and managing online databases and surveys.^36,37^ Additional questions were added regarding sibling services; those results will be reported in another article.

After institutional review board approval from both Vanderbilt University and University of Maryland, a list of potential participating institutions was created. Institutions considered for this study consisted of primary teaching hospitals (*N* = 77) that participated in the initial study, previously identified through the National Association of Children's Hospitals and Related Institutions (NACHRI) website.^16^ Two trained research assistants (RA), both PhD students and experienced in palliative care research studies, called each hospital to identify the best person to complete a survey regarding legacy activities offered to pediatric patients. They asked to speak with hospital (1) pediatric palliative care directors, (2) child life directors, (3) nursing directors, or (4) child life specialists, respectively, to further explain study details.

For individuals verbally agreeing to participate, the RA immediately e-mailed the electronic REDCap survey link. The electronic survey included an introduction that described the goal of the project, defined the term legacy-making (“…actions or behaviors aimed at being remembered,” and included examples (“…memory books, handmolds, songwriting, artwork, photos, and videos.”). The introduction also explained that they survey would take ∼15 minutes to complete, responses would be anonymous, and results would only be reported in aggregate to maintain confidentiality. Participants received e-mail reminders to encourage participant response. Participants who completed the survey received a $25 Starbucks electronic gift card.

Data collection occurred over four months (November 2019 to March 2020). Enrollment was suspended in March 2020 to respect participant burden due to COVID-19. Based on preliminary data, including similar geographic distributions of the current sample to our previous sample, researchers closed the study and proceed with analysis. Using the distributions of responses to each question observed in the prior study as the expected values for the current distributions under the null hypothesis, chi-square goodness-of-fit tests were used to test for differences between those distributions. An alpha of 0.5 (*p* < 0.05) was used for statistical significance. Qualitative content analysis identified recurrent themes within open-ended feedback. Two trained coders began the process with immersion (i.e., repeatedly reading transcripts), then clustered similar ideas/excerpts to inform preliminary categories, reviewed and revised the coding scheme, and repeated this process until no new themes emerged (i.e., saturation) and consensus was reached.

## Results

### Participants

All 77 hospitals were contacted at least once. Of those, 2 actively refused, and 18 passively refused (did not respond to voicemails). Reasons for refusal included (1) difficulty scheduling a phone meeting and (2) hospital policy prohibiting staff from sharing information requested in the survey. Three verbally agreed but never completed the survey. In total, 54 of 77 (70.1%) hospitals participated. The majority of participants were child life specialists (*n* = 43; 79.6%) from hospitals in the Northeast (*n* = 22; 40.7%), with <100 beds (*n* = 20; 37.0%), and with pediatric palliative care teams (*n* = 36; 66.7%). See Table 1 for participant demographic information.

**Table 1.** Participant (*N* = 54) Demographic Characteristics

Role within facility	
Child life specialist	43 (79.6)
Nurse	1 (1.9)
Palliative care director	1 (1.9)
Other	9 (16.7)
No. of beds	
<100	20 (37.0)
101–150	15 (27.8)
151–200	9 (16.7)
>200	10 (18.5)
Pediatric palliative care team	
Yes	36 (66.7)
No	18 (33.3)

### Comparison of current survey with previous survey

Table 2 includes survey questions and responses. All 54 (100%) participants reported that their facility offers legacy activities to children and/or their families. Differences in distributions of legacy activities reported between the prior and current study were statistically significant in some of the areas regarding type of activities offered, department offering activities, who participates, numbers of patients participating, point in the illness trajectory that services are offered, and perceived difficulties.

**Table 2.** Counts and Percentages Describing Legacy-Making Activities

Survey item	2012 Study (n* = 77), *n (%)	2020 Study (n* = 54), *n (%)	p
**What legacy-making activities does your facility offer? (select all that apply)**			
Hand molds/handprints	75 (97.4)	54 (100)	0.732
Lock of hair	68 (88.3)	53 (98.1)	0.025
Memory book or journal	65 (84.4)	38 (70.4)	0.004
Photography	58 (75.3)	36 (66.7)	0.138
Art	47 (61.0)	35 (64.8)	0.577
Writing (letters, poetry, etc.)	44 (57.1)	22 (40.7)	0.014
Songwriting/music	35 (45.5)	30 (55.6)	0.133
Video	15 (19.5)	10 (18.5)	0.864
Other	15 (19.5)	21 (38.9)	<0.001
**What department or program offers these activities? (select all that apply)**			
Child life	73 (94.8)	53 (98.1)	0.269
Nursing	36 (46.8)	17 (31.5)	0.025
Palliative care	22 (28.6)	13 (24.1)	0.469
Other	17 (22.1)	9 (16.7)	0.341
Hospice	5 (6.5)	2 (3.7)	0.407
**Who participates in completing the activity? (select all that apply)**			
Patient and his/her family together	59 (76.6)	43 (79.6)	0.607
Staff (e.g., staff does the activity and gives the result to the family)	52 (67.5)	42 (77.8)	< 0.001
Family alone	26 (33.8)	24 (44.4)	0.095
Patient alone	25 (32.5)	22 (40.7)	0.191
Other	20 (26.0)	12 (22.2)	0.535
**What pediatric patients are offered legacy-making activities? (select all that apply)**		***n* = 53**	
Patients with any life-threatening illness	65 (84.4)	46 (86.8)	0.623
Patients with cancer	33 (42.9)	25 (47.2)	0.523
Other	24 (31.2)	19 (35.8)	0.458
Patients with neurodegenerative diseases	23 (29.9)	21 (39.6)	0.118
**Approximately how many patients at your facility participate in legacy-making activities each year?**	***n* = 74**	***n* = 52**	**<0.001**
0–10	25 (33.8)	3 (5.8)	
11–20	7 (9.5)	9 (17.3)	
21–30	17 (23.0)	10 (19.2)	
31–40	12 (16.2)	5 (9.6)	
41–50	6 (8.1)	4 (7.7)	
>50	7 (9.5)	21 (40.4)	
**What point in the illness trajectory are patients and/or families offered these activities?**	***n* = 73**		**0.013**
Before the patient dies: when cure is no longer being sought	31 (42.5)	17 (31.5)	
Other	23 (31.5)	27 (50.0)	
After a child dies	10 (13.7)	8 (14.8)	
Before the patient dies: soon after patient's diagnosis	9 (12.3)	2 (3.7)	
**Legacy-making activities are offered**	***n* = 72**	***n* = 53**	**0.679**
As a part of standard of care	70 (97.2)	52 (98.1)	
Only if requested by the patient and/or their family	2 (2.8)	1 (1.9)	
**What is the goal of these activities provided at your facility? (select all that apply)**			
To benefit bereaved families	69 (89.6)	51 (94.4)	0.246
To benefit child/patient	59 (76.6)	41 (75.9)	0.898
To benefit the family while the child is ill	53 (68.8)	42 (77.8)	0.158
Other	8 (10.4)	5 (9.3)	0.789
**How do these activities help your patients and families? (select all that apply)**			
Gives family members tangible ways to remember the deceased child	74 (96.1)	54 (100.0)	0.439
Coping strategy for family members who have experienced the death of a child	66 (85.7)	49 (90.7)	0.293
Coping strategy for child patients	58 (75.3)	37 (68.5)	0.243
Coping strategy for family members who have an ill child	57 (74.0)	46 (85.2)	0.062
Creates an opportunity for children and families to talk about death	57 (74.0)	41 (75.9)	0.756
Creates an opportunity for child patients to express themselves	54 (70.1)	42 (77.8)	0.223
Gives children opportunities to do or say something to be remembered	52 (67.5)	41 (75.9)	0.191
Other	7 (9.1)	9 (16.7)	0.052
**What do you perceive is difficult for patients or families participating in legacy-making activities? (select all that apply)**			
It is emotionally hard for families to participate (e.g., it takes hope away from families)	59 (76.6)	45 (83.3)	0.247
It is physically hard for patients to participate (e.g., they are too sick, too tired)	35 (45.5)	34 (63.0)	0.009
It is emotionally hard for children to participate (e.g., these activities make children sad)	21 (27.3)	13 (24.1)	0.603
It is developmentally difficult for children (e.g., these children do not understand what these activities mean)	16 (20.8)	13 (24.1)	0.546
Other	9 (11.7)	14 (25.9)	0.001

Totals for each survey item may be >100% as respondents were allowed to select multiple responses.

#### Types of activities

Compared with 2012, statistically significant decreases were noted in types of activities, including written expression (memory book/journal and writing). Participants reported notable increases to lock of hair activities and “other” items such as creating jewelry or heartbeat recordings/songs/teddy bears.

#### Department offering activities

Similar to 2012, child life continues to most frequently (98.1%) offer legacy activities to children and their families. Notable decreases were noted in nurses offering legacy services, now with 31.5% compared with 46.8% previously (*p* = 0.25).

#### Who participates and numbers of patients participating

Similar to prior, 100% of respondents stated that their hospital offers legacy activities with 98% providing such services as a standard of care. Patients and families together still most frequently (79.6%) participate in these activities together. Increases were seen in staff completing legacy activities for the family, now with 77.8% compared with 67.5% previously (*p* ≤ 0.001). Notable increased numbers of children are participating in legacy services at children's hospitals across the United States, now with 40% (compared with 9.5% previously) of participants reporting >50 children per year. Similarly, 5.8% (from 33.8%) report 10 or fewer children participating in legacy activities per year.

#### Point in the trajectory when services are offered

Half (50%) of respondents reported that legacy services are offered at all points throughout the illness trajectory, noted as “other” in the response options. This included before and after a patient's death.

#### Perceived difficulties

Similar to prior, respondents most frequently (83.3%) reported barriers to legacy services, including that it is emotionally hard for families to participate. Significant increases were seen in reports of it being physically hard for patients to participate, now 63% compared with 45.5% in 2012 (*p* = 0.009).

### Future research

Forty-eight (89%) participants reported that more research is needed regarding pediatric legacy activities. Of the 48 participants, 14 suggested the need to determine potential benefits of legacy services to patients and their family members. For example, one participant suggested research to explore the “benefit of legacy work prior to the death, when the child is still able to participate.” Others suggested examination of “benefits of legacy materials/activities for families” and “effects on siblings.” One participant suggested research including potential benefits of legacy services on health care providers: “I would also like to know more about the impact of facilitating legacy and end of life projects on CCLS [certified child life specialists].” Thirteen participants suggested research related to the long-term effects of legacy activities. For example, one participant suggested “follow-up with families sometime after the death of a child (six months, a year, etc.) and ask them if they were offered legacy items, did they participate, and if so, has it helped with the grieving process.” Six participants wanted research to enhance our understanding for “when is the best time to introduce [legacy] services.”

Although some suggestions for future research focused on what types of legacy activities or services were most beneficial, some participants emphasized that the mechanism of intervention effect is based on the legacy-building process rather than the product. One participant said, “This experience is about process not product… all the items play the same role in supporting the family.” Another participant shared, “The memory is in the doing, rather than in the creation of a thing. The thing is the product of an amazing experience together and a reminder of that time.”

## Discussion

This study describes and compares legacy activities currently offered in pediatric hospitals compared with approximately eight years prior, based on staff perspectives. Our results suggest that numbers of children participating in legacy activities are increasing. Because recent data suggest that fewer children are dying in the hospital than in the past,^38,39^ our results may instead reflect an increase in offering of legacy activities, perhaps to a broader subset of patients than at the prior time point. This is further supported by the breadth of answers to the question about which pediatric patients are offered legacy activities, including NICU patients, those with life-threatening traumatic injuries, those on life-support for extended periods of time, and those referred to hospice.

Despite increased participation in legacy services and perhaps offering to a broader population, staff perceived the goals of legacy activities as the same—primarily to benefit bereaved families more so than the patients themselves. All participants indicated that legacy interventions give bereaved families a tangible way to remember their deceased child. Recent data suggest that parents who participate in legacy artwork with their child before their child's death have less prolonged grief and report feeling more supported by the hospital staff.^30^ Although not statistically significant, there was a slight increase (85% vs. 74%) in respondents who felt that legacy activities were useful as a coping strategy for family members of ill children, which may also be reflective of offering these opportunities earlier in the illness trajectory before death is imminent at some institutions. Overall, the additional benefits of providing an opportunity for children and families to talk about death and helping families cope after a child's death are consistent with what has been reported as benefits of legacy artwork in qualitative interviews with parents after a child's death.^40^ Some respondents indicated that siblings participated in legacy activities and that a primary goal was to help siblings. Although the benefits of participation in legacy activities for siblings of dying children are not well known, it is clear that most siblings experience personal and/or relationship changes after the death.^41^ More research should be done in this area to determine if participation in legacy activities may also benefit siblings.

It is not clear if the process of legacy activities or the final product is most important. The majority of participants reported that children and their families complete the activity together, which allows opportunities for memory-making and conversations about death with the sick child. Parents have reported that a storytelling legacy intervention for children with cancer helped children express their feelings, suggesting the process of participating in legacy-making was important for the child.^23^ However, since the last national legacy survey,^16^ results of our current study suggest a significant increase in reports of *staff* completing legacy activities without the family's participation, as well as increased reports of *parents and siblings* completing legacy activities alone. Both of these cases exclude the child's participation, such that the child would not benefit from the process of legacy-making. This could suggest that health care professionals have recognized the potential benefits of legacy interventions for both children with serious illness as well as families when the patient cannot benefit from participation.

Important to note is the increased concerns that it is physically too difficult for children to participate. This could be due in part to increasing hospitalization rates of medically complex children^42^ and may explain the increase in staff and family-only participation. This may also explain the trends in the activities offered. Nearly all institutions offer handprints/molds and a lock of hair, neither of which requires active participation from the child. However, fewer institutions than prior indicated they are offering journaling and writing, activities that may require more engagement of the child. In summary, legacy activities can be individualized to the needs and wishes of children and families. More study is needed to help determine what patients may be most likely to benefit from active participation in the process, whereas other families may benefit more from receiving a final product created by staff, for example.

Child life departments are leading the delivery of legacy activities at nearly all institutions, with fewer institutions than prior reporting that legacy activities are offered through the nursing department. Although nurses caring for dying children frequently identify the facilitation of making memories and creating mementos as one of their roles, nurses have numerous other important roles in guiding families through their child's death.^43,44^ It may be that the actual offering of legacy activities has been delegated more completely to child life specialists, reflecting the growth of the child life profession since the prior time point. More study is needed to clearly delineate how other health care professionals (e.g., physicians, advanced practice nurses, social workers, music therapists, and chaplains) can support child life departments delivering this care at the bedside.

We acknowledge several limitations of our study. First, participants were limited to those from institutions represented in the previous sample to allow for data comparison; thus, the study does not account for the growth in children's hospitals since 2012. The large majority (nearly 80%) of participants were child life specialists, so results may not accurately reflect perceptions of other health care providers. Although we did not aim to talk with the same person at each hospital from the previous study, there is potential bias if participants read the first national survey. The majority of respondents represented hospitals located in the Northeast, thus results may not be representative of legacy services in the United States. Results also may not generalize to hospitals outside of the United States or adult patient populations. Despite these limitations, our study contributes new information to the field of pediatric palliative care and how legacy services have evolved over time.

## Conclusion

This novel study provides an updated description of legacy services offered by children's hospitals across the United States to children and their families and compares results with our prior study. Research related to legacy interventions for children has substantially advanced over the past decade, with more children receiving services. Future research should evaluate the impact of legacy interventions on family members' (e.g., parents and siblings) experiences at end of life and in bereavement. Studies should also determine the best time in the illness trajectory to offer legacy services and their impact to families over time. Extension of our study is needed to determine types of legacy interventions offered through nonhospital settings, such as pediatric hospice or other pediatric palliative care organizations. If less children are dying in hospitals, it is important for providers to offer this standard of care to children dying in their homes. Longitudinal designs that examine outcomes from predeath through bereavement are needed to examine the short- and long-term effects of legacy interventions. Mechanistic studies need to be conducted to move intervention work forward, and physiological measures can be considered in addition to psychosocial measures.

Our findings illustrate that legacy services are a sustained and growing component of palliative care being offered to children with serious illness and their families. This study documents that legacy-building has moved from an anecdotal hospital service to an empirical-based field of research over the past decade. Palliative care providers can partner with researchers to continue advancing the science and increase recognition of this important inquiry of research. Health care providers should continue to offer legacy opportunities to children with serious illness and their families across a wide array of settings and illness contexts, including earlier in the illness trajectory and in hospital and home-based environments. Providers can educate patients, family members, and health care professionals that legacy services are not only for children near end of life, but also those who will be cured and live long healthy lives. More research is needed to translate evidence-based legacy interventions to pediatric palliative care practice.

## Abbreviation Used

RA: research assistants
